# Community engaged research to measure the impact of COVID-19 on vulnerable community member’s well-being and health: A mixed methods approach

**DOI:** 10.1007/s00508-022-02113-z

**Published:** 2022-12-05

**Authors:** Amelia K. Barwise, Jason Egginton, Laura Pacheco-Spann, Kristin Clift, Monica Albertie, Matthew Johnson, Sarah Batbold, Sean Phelan, Megan Allyse

**Affiliations:** 1 Division of Pulmonary and Critical Care Medicine, Mayo Clinic, 200 First Street SW, 55905 Rochester, MN USA; 2 Program in Biomedical Ethics Research, Mayo Clinic, Rochester, MN USA; 3 Robert D. and Patricia E. Kern Center for Science of Health Care Delivery, Mayo Clinic, Rochester, MN USA; 4 Quantitative Health Sciences, Mayo Clinic, Jacksonville, FL USA; 5 Center for Individualized Medicine, Mayo Clinic, Jacksonville, FL USA; 6 Center for Health Equity and Community Engagement Research, Mayo Clinic, Jacksonville, FL USA; 7 Mayo Clinic Alix School of Medicine, Rochester, MN USA; 8 Division of Health Care Delivery Research, Mayo Clinic, Rochester, MN USA; 9 Department of Obstetrics & Gynecology, Mayo Clinic, Rochester, MN USA

**Keywords:** SARS-CoV‑2, Pandemic, Community-based participatory research, Mixed methods research, Health disparities

## Abstract

**Background:**

The COVID-19 pandemic has exacerbated existing income inequality and health disparities in the United States (US). The objective of this study was to conduct timely, community-engaged research to understand the disproportionate impact of the COVID-19 pandemic on historically under-resourced communities with the goal of improving health equity. The initiative focused on priorities identified by Community Health Needs Assessments (CHNA) conducted every 3 years per Federal funding requirements. These were access to healthcare, maternal/child health, obesity/food insecurity/physical activity, and mental health/addiction.

**Methods:**

In the first three quarters of 2021, we developed and employed mixed methods in three simultaneous phases of data collection. In phase 1, we used purposive sampling to identify key informants from multiple stakeholder groups and conducted semi-structured interviews. In phase 2, we held focus groups with community members from historically marginalized demographics. In phase 3, we developed a survey using validated scales and distributed it to diverse communities residing in the geographic areas of our healthcare system across four states.

**Conclusion:**

Healthcare systems may use the methodology outlined in this paper to conduct responsive community engagement during periods of instability and/or crisis and to address health equity issues. The results can inform sustainable approaches to collaborate with communities to build resilience and prepare for future crises.

## Introduction

The 2020 COVID-19 pandemic brought into sharp focus the widespread social and economic inequity in the United States (USA) and the heightened inability of existing economic and health institutions to serve vulnerable communities [1, 2]. Racial, ethnic, and income disparities have been documented in rates of COVID-19 exposure, infection, morbidity, and mortality as well as access to and uptake of testing and vaccination [3–5]. For many, social determinants of health and concerns about job losses, hunger, and homelessness have overshadowed concerns about viral transmission and prevented individuals from implementing basic infection control measures [6–9].

Healthcare systems that benefit from tax-exempt status are required by the Patient Protection and Affordable Care Act to conduct a Community Health Needs Assessment (CHNA) every 3 years to assess the health needs of surrounding communities. Although CHNAs are important to track health trends geographically and in response to local public health measures, they have limitations [10]. As they are not geographically standardized, CHNAs differ widely on a county level, including variations in methodology, health priorities, sample size, and definition of key terms [11, 12]. Within our healthcare system the CHNAS at each of the sites are conducted differently and by different types of groups and employees. For larger health systems with multiple locations, it can be challenging to integrate multiple, diverse, health assessments into meaningful action on local health and engagement. In addition, CHNA exercises are conducted on a fixed schedule dictated by Federal tax filings. It can be challenging to pivot this infrastructure to respond rapidly to an unfolding crisis such as the COVID-19 pandemic of 2020–2021. Finally, while the CHNA process does require health systems to identify actions to be taken in response to CHNA findings, there is no mechanism for monitoring and documentation that such actions have been taken or have been successful or had a “collective impact” [13, 14]. The CHNA is thus a less effective process for providing highly responsive, short-term information feedback on the immediate impact of health crises such as the COVID-19 pandemic and some have argued that to strengthen CHNAs a more community-based participatory research approach would be helpful [15].

Community engaged research (CEnR) is designed to facilitate the translation of scientific discovery into a reduction in health disparities by understanding stakeholder and community needs and “to allow scholars and community members to collaborate to identify and implement solutions to community problems” (Showstack et al.) [16]. The CEnR approaches have many benefits including influencing public health initiatives and health outcomes outside the clinic [17, 18]. For this reason, CEnR is an important complement to traditional translational approaches in addressing health equity issues in communities. In early 2021, at the request of our institution, we initiated a healthcare system-wide initiative to complement CHNAs efforts by implementing a rapid response approach to assessing health needs and resources in ways that cut across multiple jurisdictions and geographic areas.

The overall objective of this study was to conduct empirical CEnR to understand the specific and disproportionate impact of the COVID-19 pandemic on historically marginalized and under-resourced communities, identify novel approaches to address inequity and explore current community assets that mitigate the worst impacts of the pandemic [19–21]. The purpose of this paper is to provide a methodological blueprint for other healthcare systems considering responsive CEnR to understand the needs of their catchment population and support initiatives that advance health equity.

## Methods

### Study setting and design

We used the priority health domains from the most recent CHNA reports to identify topic areas previously prioritized by local community and public health departments in the three counties in which our system has a primary presence. We met with CHNA teams, including internal public engagement staff and members of local departments of health, to assess the status of the CHNA process in each region, coordinate content, and attempt to avoid any duplication of effort. These meetings continued through the course of the initiative.

After synthesis, the four combined domains we identified were access to care, maternal/child health, obesity/food security/physical activity, and addiction and mental health. Based on our review of the CHNA reports, which focused on general findings during non-COVID times about the general catchment area population, we believed a more detailed understanding of the specific needs of vulnerable populations was warranted to provide guidance for action on health equity. We considered our study a “deeper dive” into identified issues of concern.

Data collection occurred between March and November of 2021. We conducted a multi-site healthcare system-wide initiative engaging with communities within the catchments areas of the three largest hospitals of Mayo Clinic as well as the Mayo Clinic healthcare system. Mayo Clinic tertiary care academic medical centers are located in Rochester, (MN) Jacksonville, (FL) and Scottsdale, (AZ). The healthcare system spans several states including Minnesota and Wisconsin.

The study employed mixed quantitative and qualitative methods in three phases. Phase 1 involved qualitative key informant interviews, phase 2 involved focus groups and phase 3 employed an anonymous, online survey. The qualitative phases of the study were approved as minimal risk by the Mayo Clinic institutional review board (IRB). The quantitative phase was conducted entirely by an external, professional survey research company. As verified by research compliance, Mayo Clinic had no participant contact, was therefore not engaged in human subject research, and this phase was not eligible for IRB review.

### Phase 1: key informant (KI) interviews

#### Participants and recruitment

We used purposive sampling to identify key informants (KI) from multiple stakeholder groups including county public health officials, safety net medical providers, community leaders, non-profit organization executives and staff, and health system leadership. Individuals were asked to participate based on the likelihood that they would have useful insights for our study and were often suggested by our public engagement leaders who spearhead the CHNA. We reached out to stakeholders via email or telephone to outline the nature of the study and participation requirements. Consent forms were automatically emailed to those who agreed to participate and completed via DocuSign (DocuSign, San Francisco, CA, USA) prior to the interview [22]. We interviewed 24 KIs.

#### Interview guide

The interview guide was developed by the broader study team and included questions that explored the mission of the organization within which the KI was working, the communities the organization served, the health challenges facing those communities, current efforts to address the needs and assets, and research needed to amplify current work. Other questions explored the specific effect of COVID-19 in those communities, how services were modified to meet needs, and suggestions for efforts and research to support and prepare communities for future similar events as well as the organization’s relationship with local health institutions and current collaborative initiatives.

#### Data collection

Following consent, study team members conducted one-on-one, semi-structured interviews. Interviews were scheduled for up to 60 min and were conducted via online video conferencing [23]. Interviews lasted 30–40 min in general. Financial remuneration was not deemed appropriate by the study team and the IRB concurred.

### Key informant qualitative analysis

All KI interviews were digitally recorded. Audio recordings were transcribed verbatim by a HIPAA-concordant professional transcription firm and de-identified prior to analysis.

Interview data were organized and analyzed using the framework analytic approach [24]. This accessible and efficient approach was chosen for several reasons. Some members of our research team were experienced qualitative researchers, and others were less experienced. Under the leadership of our experienced coders the framework method provided a systematic and flexible approach to analyzing our data. The index paraphrasing is readily auditable allowing any research team member to participate in sense-making of the large dataset. Additionally, given the multidisciplinary nature of our study team the framework approach provided a helpful structure for cross-disciplinary analysis using matrices we developed.

For the KI qualitative phase, coders collaborated on each phase of analysis from initial content paraphrasing and indexing to codebook development and exemplar (quote) retrieval.

### Phase 2: focus groups

#### Participants and recruitment

We conducted focus groups (FGs) with individuals in all three regions who represented different residential, professional, and social communities. We distributed flyers in Spanish and English through social media and community organizations. The purpose of the study, risks and benefits, and the option to decline any question and/or withdraw at any time were outlined to participants before scheduling. In addition, we worked collaboratively with local departments of health who were conducting focus groups with similar topic areas for their own purposes. Ultimately, our group conducted 16 focus groups and negotiated for data sharing of 31 more.

#### Moderator guide for focus groups

The moderator guide was developed by the broader study team and community partners and included questions about hopes following COVID-19, the impact of COVID-19 on access and use of health care, the personal impact of COVID-19 on other aspects of life, how COVID-19 affected vulnerable communities and the availability of resources and potential assets need to support those communities, as well as thoughts about the vaccine.

#### Data collection

The FGs were scheduled for and lasted approximately 60 min and were conducted via either online videoconference or in person (for participant communities that lacked broadband access), audio-recorded, de-identified, and transcribed. The moderator began the activity by outlining the elements of consent which had been shared prior to scheduling with participants and they were asked to confirm consent verbally. To the greatest extent possible, ethnically, and racially congruent moderators were used in FGs and two were conducted by bilingual moderators in Spanish. Participants received a modest amount of remuneration.

### Focus group qualitative analysis

All FGs were digitally recorded, and the audio recordings were transcribed verbatim by a HIPAA-concordant professional transcription firm and de-identified prior to analysis. The FGs conducted in Spanish were professionally transcribed and translated by a licensed medical translator. Data from FGs conducted by our team and those conducted by others were stored and analyzed in parallel using identical coding structures but were not combined into one dataset.

As with the KI data, FG data were organized and analyzed using the framework analytic approach, although the two datasets were analyzed separately [24]. For this qualitative phase, two trained coders collaborated on each phase of analysis from initial content paraphrasing and indexing to codebook development and exemplar (quote) retrieval. Coding was guided deductively by CHNA domains, as well as the moderators’ guide built around those constructs. Coding was also left open to inductive findings as they arose during the coding process.

### Phase 3: survey

A community survey was developed to assess stressors, resources, well-being, and health care access and outcomes associated with the COVID-19 pandemic. The survey was fielded through organizations serving the communities surrounding Mayo Clinic in Minnesota, Wisconsin, Florida, and Arizona. These organizations were those that had worked with the healthcare system in previous CEnR or through outreach efforts.

#### Survey instrument

The survey was developed with the input of community and scientific leaders and stakeholders. First, relevant constructs were selected based on a scan of the scientific literature around stress and resilience, disaster recovery, and COVID-19. The construct list was augmented with input from key stakeholders in the Mayo Clinic Center for Health Equity and Community Engaged Research (CHCR), the Mayo Clinic Comprehensive Cancer Center, and three community advisory boards (CAB). Measures representing relevant constructs were selected from published literature based on published construct and predictive validity and reliability. Measures with face-validity were created by the study team when no appropriate scale could be located. Some scales were shortened to reduce respondent burden by selecting items with highest factor loadings or item-scale correlations or based on study team evaluation of content if psychometric properties were unpublished. Consideration was also given to ensuring subscales were represented by multiple items. Survey measures assessed facets of COVID-19 testing, vaccination and strategies adopted to mitigate the risk of infection as well as resilience, coping, isolation, community, and support. The survey also explored the impact of COVID-19 on medical care, ability to access healthy food and physical activity, physical and mental health, and opinions about telehealth and clinical trials. The survey was extensively pilot tested and we solicited comments and suggestions from CABs, existing panels of research scientists and broader panels of community engagement researchers across the institutions and volunteers [25]. The survey was also translated into Spanish by a licensed medical translator and tested by bilingual English-Spanish speakers.

#### Participants and sampling strategy

The survey link was disseminated through existing networks of community partners and health ministries including social media and email lists for a period of 8 weeks in summer and fall of 2021. Recruitment material also included advertisements in community gathering locations. We contracted a professional research firm to host, accrue, and de-identify data. Participants who completed the survey had the option to receive a small financial remuneration via email.

### Data analysis plan

Raw data were “cleaned” using standard practices including humanistic and heuristic approaches that involve inclusion of survey completion time, originating IP address, assessment of correlated variables and plausible associations, and attention-check item response, to reduce the likelihood of including fraudulent or duplicate responses [26]. Analysis is underway and has consisted of three phases: 1) exploratory factor analyses to ensure scale items load to their intended scales, including calculation and reporting of psychometric properties, 2) univariate descriptive analyses, including calculating means, standard deviations and scale and item distributions and weighing sample analyses appropriately to represent population estimates for the three geographic regions and 3) bivariate and multivariate models to examine relationships between variables. As we leveraged social media for disseminating the survey, we have no mean of calculating the response rate; however, we can report that after data cleaning the completed dataset included 3445 English language responses and 146 Spanish language responses. Responses are proportionate to the geographic population of the area surveyed.

## Discussion

The purpose of this initiative is to understand the impact that the COVID-19 pandemic has had on marginalized and minority communities in three geographically and demographically distinct areas, how communities are coping, and how health systems can be better partners in building resilience in preparation for future pandemics. Using the results of community engagement exercises like these, health systems can strengthen their status as anchor institutions by enhancing their impact in improving health equity and sustainable solutions to mitigate health disparities [27, 28].

This paper outlines the methodological approaches we used to undertake this initiative within a limited timeframe, across multiple states and sites, and using mixed methods to garner insights and data for a healthcare system-wide evaluation of the needs and assets of the catchment areas surrounding our institutions. We successfully and safely collected qualitative data using a video conferencing platform, largely avoiding in person interviews and FGs during the COVID-19 pandemic and allowing our research to occur while adhering to COVID-19 guidelines. Furthermore, we strategically recruited our FG participants based on the shared priority topics identified in the most recent CHNAs using CEnR to explore these topics in more depth and with the goal of developing initiatives to address the issues raised.

This work included Spanish language FGs, and distribution of a survey translated into Spanish thus increasing the reach of our research efforts and the generalizability of our findings to diverse populations. Our survey largely utilized validated scales from existing surveys, and we sought feedback from CABs and other volunteers about readability and relevance before disseminating [29, 30]. Unlike many surveys, we did not use a survey panel but distributed the surveys throughout our catchment area communities. Although this was a “convenience sample”, the large numbers and diverse demographics of our participants, which are reflective of the general population in the regions surveyed, are a strength of this work. Other strengths include the use of the framework method for qualitative analysis. This approach can be deployed by other study teams who have limited qualitative experience and resources, providing efficiency, accessibility, and a robust audit trail to multidisciplinary teams. Furthermore, unlike much qualitative research analysis, the framework method does not require specialized, licensed, and often expensive software such as NVivo (QSR Intl Inc; Burlington, MA, USA). Therefore, the framework method can reduce barriers to conducting this type of work and can be utilized with access to low cost and readily accessible software such as excel.

We hope that this paper will serve as a methodological resource for other healthcare systems and institutions who want to conduct CEnR and respond to their community needs. While we have tried to provide helpful details for future researchers, we recognize the disparate resources, community partnerships and research infrastructure among healthcare institutions and that this may affect the applicability of our methodology for CEnR in some locations; however, with adaptation to local circumstances, we believe that this form of multilayered, community-engaged, mixed methods approach allows a diversity of data types that can assist in triangulation around the true needs of communities.

Using an online platform for FGs provided great flexibility for most participants and widened the breadth of people we could recruit; however, connectivity issues and technical proficiency may have deterred some from enrolling in the study or unexpectedly limited their participation. We attempted to ameliorate this restriction by conducting in person FGs for certain populations.

We faced several challenges during our study. Despite the perception that recruitment during a pandemic could be difficult we did not have issues with recruiting and scheduling KI interviews or FGs; however, one issue which we did note was the significant number of investigators and organizations also seeking community feedback during this time. This required us to negotiate and engage in frequent dialogue with others to avoid duplication of efforts and to promote collegial collaborations to maximize our research output and impact. The qualitative data-sharing we did with local departments of health is an example of fruitful collaboration. Our efforts included regular meetings with our CHNA colleagues to discuss recruitment efforts and further discussions as they planned the next CHNA effort.

The importance of CEnR cannot be overstated and its potential impact for developing solutions during a crisis such as COVID-19 remain relevant [31, 32]. Those in health care leadership roles should consider the disproportionate impact of the COVID-19 crisis on historically under-resourced and marginalized communities [33]. We hope that work like this will provide rigorous data to support future endeavors to address those needs and this paper provides a robust roadmap for other institutions interested in engaging with their catchment area communities to advance health equity.
